# Identification and validation of EMT-immune-related prognostic biomarkers CDKN2A, CMTM8 and ILK in colon cancer

**DOI:** 10.1186/s12876-022-02257-2

**Published:** 2022-04-16

**Authors:** Ning Kang, Xiaoli Xie, Xue Zhou, Yijun Wang, Shengxiong Chen, Ran Qi, Ting Liu, Huiqing Jiang

**Affiliations:** 1 Department of Gastroenterology, The Second Hospital of Hebei Medical University, Hebei Key Laboratory of Gastroenterology, Hebei Institute of Gastroenterology, Hebei Clinical Research Center for Digestive Diseases, No. 215, Heping West Road, Shijiazhuang, 050000 Hebei Province China; 2 Department of Hepatobiliary Surgery, The Second Hospital of Hebei Medical University, Shijiazhuang, 050000 Hebei Province China; 3 Department of General Practice, The Second Hospital of Hebei Medical University, Shijiazhuang, 050000 Hebei Province China

**Keywords:** Colon cancer, Epithelial-mesenchymal transition, Immune, Bioinformatics analysis, Biomarker, Prognosis

## Abstract

Colon cancer (CC) is a disease with high incidence and mortality rate. The interaction between epithelial-mesenchymal transition (EMT) and immune status has important clinical significance. We aim to identify EMT-immune-related prognostic biomarkers in colon cancer. The GEO2R and GEPIA 2.0 were utilized to calculate the differential expression genes between CC and normal mucosa. Immport, InnateDB and EMTome databases were used to define EMT-immune-related genes. We conducted batch prognostic analysis by TCGA data. The expression patterns were verified by multiple datasets and lab experiments. GEPIA 2.0 and TIMER 2.0 were utilized to analyze the correlation of the hub genes with EMT markers and immune infiltration. GeneMANIA, STRING, and Metascape were used for co-expression and pathway enrichment analysis. Finally, we established a signature by the method of multivariate Cox regression analysis. CDKN2A, CMTM8 and ILK were filtered out as prognostic genes. CDKN2A and CMTM8 were up-regulated, while ILK was down-regulated in CC. CDKN2A was positively correlated with infiltration of macrophages, Th2 cells, Treg cells, and negatively correlated with NK cells. CMTM8 was negatively correlated with CD8+ T cells, dendritic cells, and NK cells. ILK was positively correlated with CD8+ T cells and dendritic cells. Moreover, CDKN2A, CMTM8 and ILK were significantly correlated with EMT markers. The three genes could participate in the TGF-β pathway. The prognosis model established by the three hub genes was an independent prognosis factor, which can better predict the prognosis. CDKN2A, CMTM8 and ILK are promising prognostic biomarkers and may be potential therapeutic targets in colon cancer.

**Supplementary Information:**

The online version contains supplementary material available at 10.1186/s12876-022-02257-2.

## Introduction

Colon cancer (CC) is a major health problem worldwide and the second-leading cause of cancer-related deaths [1]. The incidence and mortality of colorectal cancer were approximately 10.2% and 9.2% respectively [2]. Although the emerging treatments of CC occur, the prognosis is still poor [3, 4]. Therefore, it is necessary to identify prognostic biomarkers and therapeutic targets.

Epithelial-mesenchymal transition (EMT) is a cellular biological process in which epithelial cells obtain mesenchymal phenotype after epithelial characteristics are down-regulated through specific procedures [5]. EMT plays a significant role in CC progression by contributing to cell migration and tumor metastasis, promoting the occurrence of tumor stem cells characteristics, reducing apoptosis and senescence, facilitating drug resistance [6]. Another important factor in CC progression is the immune microenvironment. An effective immune response can eliminate malignant cells. However, colon cancer cells can escape from immune surveillance by multiple mechanisms, such as deficiencies in antigen presentation mechanisms, up-regulation of immune checkpoints, and recruitment of immunosuppressive cells [7]. These phenomena result in impaired function of immune cells, invalidation of anti-tumor responses, and ultimately tumor progression and metastasis [8].

Previous studies have found that EMT may interact with immunosuppression directly or indirectly [9–11]. Some studies also found that tumor cells and immune cells could respond to similar stimuli by activating alike programs, leading to the occurrence of EMT and the infiltration of immunosuppressive cells. For example, one of the most studied stimuli is TGF-β, which is a potent inducer of EMT [12]. In addition, it inhibits the infiltration of immune cells and promotes the differentiation of suppressed or exhausted immune cells [13]. If the immunosuppression and EMT occur together, the survival rate of colon cancer patients may be decreased markedly. So, the EMT-immune-related biomarkers are important.

More and more researches focused on the interaction between EMT and immune status. Moreover, some related biomarkers were explored. SNAI1, an EMT-related gene, is associated with immune infiltration and can be used as a prognostic biomarker in gastrointestinal cancers [14]. Gao et al. revealed that SLC2A3 plays a significant role in the progression of colorectal cancer by affecting EMT and immune response. Furthermore, SLC2A3 may regulate the expression of PD-L1 [15]. An EMT signature performs well in predicting the overall survival, and the model is associated with immune infiltration, proving its potential role in regulating immune status [16]. Additionally, other EMT-immune-related biomarkers, for example, LIPH, CDH11, SETBP1, and so on, also have prognostic value in different cancers [17–23]. However, they are far from sufficient, more biomarkers should be identified and verified.

According to the above information, when EMT and immunosuppression occur in a patient with CC, the risk of tumor progression and metastasis will be increased compared with others, which may affect the prognosis. As a result, this study identified and validated the EMT-immune-related prognostic biomarkers named CDKN2A, CMTM8, ILK by bioinformatics method. Moreover, the three genes were related to regulating the process of EMT and immune infiltration. Therefore, CDKN2A, CMTM8, ILK may serve as good prognostic biomarkers and potential therapeutic targets.

## Materials and methods

### Identifying the EMT-immune-related differential expression genes

The dataset GSE10950 in the GEO (https://www.ncbi.nlm.nih.gov/geo/) database [24] contains 24 cases of colon cancer and matched normal tissues. Differentially expressed genes (DEGs) between normal and CC were calculated by GEO2R, |logFC|> 1 and adjust *P* value < 0.05 were the screening criteria. With the same criteria, DEGs between normal (GTEx) and CC (TCGA-COAD) were calculated by GEPIA 2.0 online tools (http://gepia2.cancer-pku.cn/index.html) [25]. Next, we defined immune-related genes by InnateDB (https://www.innatedb.com/) [26] and Immport (https://immport.org/shared/home) databases [27]. EMT-related genes were defined by EMTome (www.emtome.org) [28].

### Screening genes by prognosis and clinical correlation analysis

The RNA-seq count data and clinical data of colon adenocarcinoma were downloaded from The Cancer Genome Atlas (TCGA) database (https://portal.gdc.cancer.gov/) [29]. After normalizing the count data to RPKM (Reads Per Kilobase of exon model per Million mapped reads) and combining with clinical data, 338 cases were finally included in the analysis. We used “survival” [30] and “survminer” packages [31] of R 4.1.0 to analyze the prognostic value of EMT-immune-related DEGs. We validated the prognostic value of the three genes by using GSE39582, GSE24511 and GSE29621 repectively. Then we built receiver operating characteristic curves (ROC curves) and conducted the correlation analysis with clinical and pathological indicators.

### Expression analysis and validation of CDKN2A, CMTM8, and ILK by multiple databases and scRNA-seq data

GSE10950 datasets, GEPIA 2.0 database, and Oncomine database (www.oncomine.org) [32] were utilized to conduct differential expression analysis of hub genes. Furthermore, single-cell transcriptome dataset GSE166555 in the GEO database was enrolled in the analysis. We chose three cases of colon cancer and matched normal tissues to build a “Seurat” object [33]. Finally, 15,428 cells were selected (Criteria: RNA Features > 500 and 1000 < RNA Counts < 20,000). Then we normalized the data by the “LogNormalize” method, calculated the highly variable genes, and homogenized according to the sample sources. Next, principal component analysis (PCA) calculation was performed. The first 20 PCs were used for cluster analysis, and the t-SNE method was used for cluster visualization (resolution = 0.3). Then marker gene calculation was carried out and the “CellMarker” method was used to annotate cell types.

### Expression validation of CDKN2A, CMTM8, and ILK by immunohistochemistry experiments

In order to verify the protein expression of CDKN2A, CMTM8 and ILK, we collected 24 paired paraffin-embedded tissues of colon cancer and adjacent tissues from the pathology department. All the cancer patients in our study were primary colon cancer and without prior treatments. For conducting the experiments, we used Anti-CDKN2A/p16INK4a (Servicebio GB111143), Anti-CMTM8 (Proteintech 15039-1-AP), and Anti-ILK (Proteintech 12955-1-AP). All the sections were deparaffinized in xylene and rehydrated in ethanol. We then used sodium citrate antigen retrieval solution (Solarbio) for heat-mediated antigen retrieval. After blocking by 3% H_2_O_2_ (30 min) and normal goat serum (1 h), the tissue sections were incubated with CDKN2A (concentration 1:200), CMTM8 (concentration 1:50) and ILK (concentration 1:50) antibodies overnight (4℃). Then we stained the tissue sections with the secondary antibody (ORIGENE SP-9001) for 20 min at room temperature. Next, horseradish labeled streptavidin (ORIGENE SP-9001) was utilized to incubate tissues for 15 min. We utilized diaminobenzidine (ORIGENE ZLI-9018) for peroxidase reaction, and the slides were stained with hematoxylin (LEAGENE). CDKN2A and CMTM8 were mainly located in the cell nucleus, while ILK was in the cell nucleus and cytoplasm. Integrated optical density (IOD) of all tissues was calculated by Image-Pro Plus 6.0 software and then the statistical analysis was conducted by Graphpad Prism 7.0. The studies involving human participants were reviewed and approved by the institutional review board of the second hospital of Hebei Medical University. The patients provided their written informed consent to participate in this study.

### The immune infiltration analysis and the EMT markers correlation analysis of CDKN2A, CMTM8, and ILK

We used R language to conduct immune-related analysis on TCGA-COAD data by the “CIBERSORT” method. The overall immune infiltration status of all the samples was analyzed. Moreover, the “Gene” module of TIMER 2.0 (http://timer.comp-genomics.org/) [34] was used to evaluate the correlation between the expression level of hub genes and the immune infiltration. Then, we utilized the “Gene_corr” module to analyze the expressional correlation between the hub genes and the classical immune cell markers. Furthermore, their correlation with the immune checkpoints was analyzed with the same method. GEPIA 2.0 online database was utilized to conduct correlation analysis between the hub genes and the traditional EMT markers by the “spearman” method.

### The co-expression and pathway enrichment analysis of CDKN2A, CMTM8, and ILK

By STRING database (https://string-db.org/) [35], we regulated the “max number of interactors to show” to “1st shell no more than 50” in order to carry out the co-expressional analysis of the hub genes. We displayed the interactions of the genes by GeneMANIA (http://www.genemania.org) [36]. Moreover, we conducted the pathway enrichment analysis of the genes by the Metascape database (http://metascape.org) [37] and showed the top 20 items.

### Establishing the ceRNA network in regard to CDKN2A, CMTM8, and ILK

To further analyze the underlying regulating mechanisms of CDKN2A, CMTM8 and ILK, we estabilshed the ceRNA network. miRNA-mRNA and lncRNA-miRNA interactions were obtained from starBase v3.0 (http://starbase.sysu.edu.cn/) [38]. The screening criteria of miRNAs were: refine results with high stringency of CLIP Data: 1, refine results with used predicting program: 4, explore the interaction of miRNA-mRNA with pan-cancer analysis: 1. The screening criteria of lncRNAs were: refine results with high stringency of CLIP data: 3, explore the interaction of miRNA-lncRNA with pan-cancer analysis: 5. The lncRNA-miRNA-hub genes’ network was visualized by Cytoscape 3.8.2 software.

### Mutation, Copy number variation, and Methylation analysis of CDKN2A, CMTM8, and ILK

We analyzed the immune infiltration changes by mutations and copy number variations (CNV) of hub genes by the TIMER 2.0 database. The database cbioportal (www.cbioportal.org) [39] was used to analyze the effect of hub genes’ mutations and CNV on phenotype and prognosis. Totally 139 cases consist of 8 patients with the mutation were enrolled. The samples were from 2 datasets, “CaseCCC, PNAS 2015” and “CPTAC-2 Prospective, Cell 2019”. Moreover, we took advantage of MethSurv (https://biit.cs.ut.ee/methsurv/) [40] to analyze the effect of methylation in prognosis. “Single CpG” module was chosen to do the analysis, and the “split by” option chose “median”.

### Construction of EMT-immune-related signature by CDKN2A, CMTM8 and ILK

By the results of multivariate Cox regression analysis, the riskscore of each patient was calculated by the following formula: Riskscore = Expression (CDKN2A) * Coef (CDKN2A) + Expression (CMTM8) * Coef (CMTM8) + Expression (ILK) * Coef (ILK). Then all patients were divided into high-risk and low-risk groups by the median of riskscore. The Kaplan–Meier curve and ROC were analyzed by the “survival”, “survminer”, and “timeROC” packages [41]. These results could assess the accuracy of the riskscore. The univariate and multivariate Cox regression analyses were used to determine the independent prognosis factors. A nomogram was used to predict the survival probability of CC patients by “rms” package [42].

## Results

### EMT-immune-related differential expression genes were filtered out

**Fig. 1:**
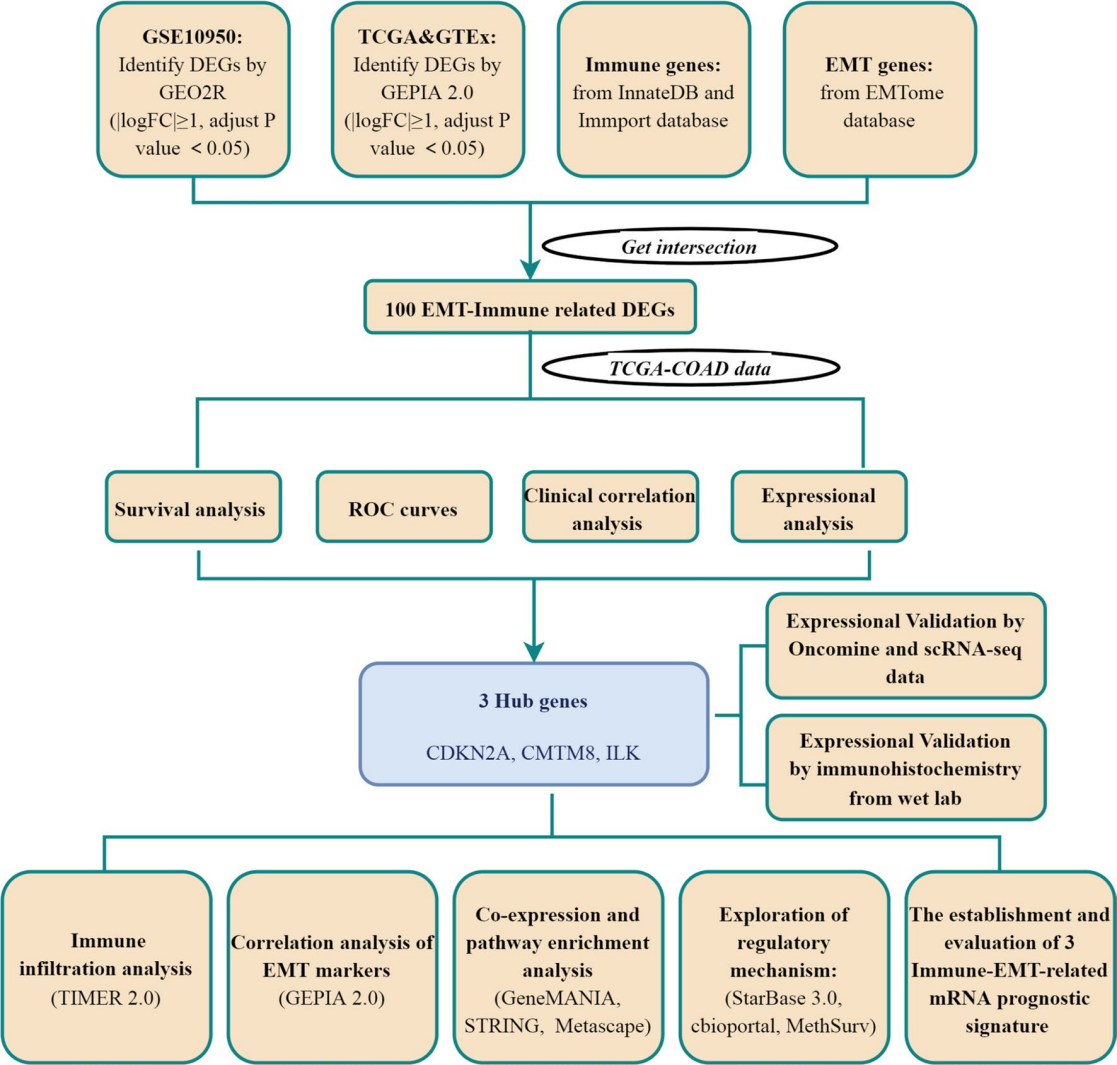
Flowchart for screening biomarkers in colon cancer

**Fig. 2:**
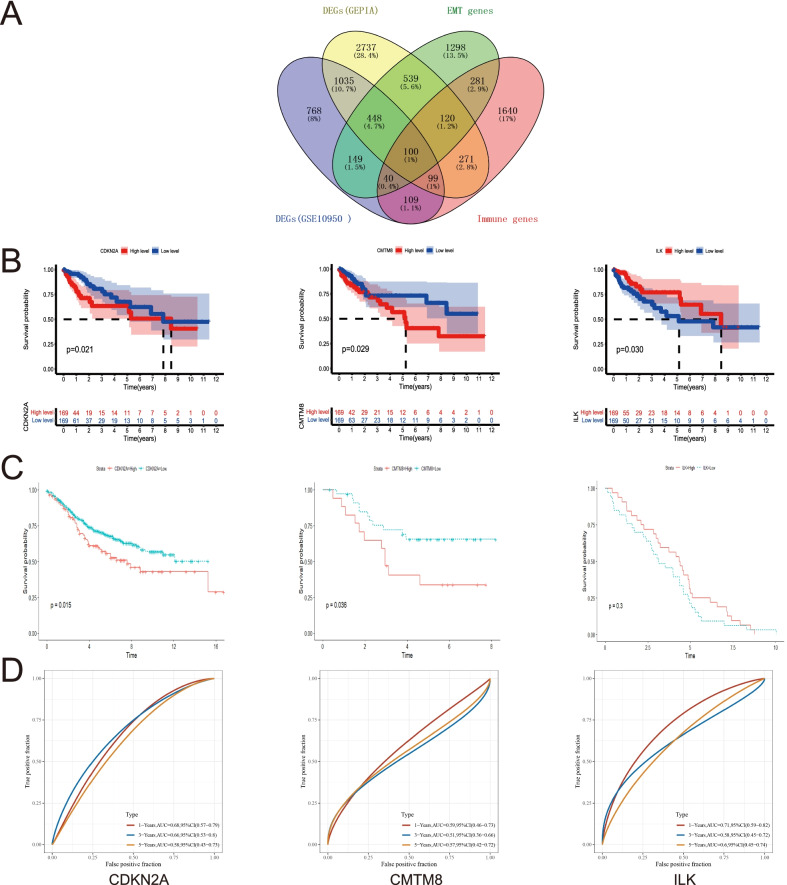
Identifying the EMT-immune-related prognostic DEGs. **A** Venn plot displaying the identification of EMT-immune-related DEGs. **B** Kaplan–Meier survival analysis of CDKN2A, CMTM8 and ILK in TCGA-COAD data. **C** Kaplan–Meier survival analysis of CDKN2A, CMTM8 and ILK in GSE39582, GSE24551, GSE29621 respectively. **D** ROC analysis of CDKN2A, CMTM8 and ILK in CC

Recent studies have found that EMT and tumor immunity are closely related, moreover, they are considered to be driving factors in tumor progression. The genes involved in both biological processes may be significant. So we screened EMT-immune-related differential expression genes. The workflow is exhibited in Fig. 1. In total, 2748 DEGs were calculated by the GSE10950 dataset, and 5349 DEGs were obtained by GEPIA 2.0 database. We defined 2660 immune-related genes by InnateDB and Immport databases. 2975 EMT-related genes were defined by the EMTome database. The intersection among the DEGs of GSE10950 and GEPIA 2.0, immune-related genes, and EMT-related genes were 100 genes, which were defined as EMT-immune-related DEGs (Fig. 2A).

### CDKN2A, CMTM8, ILK were genes with prognostic value among 100 EMT-immune-related DEGs

By single-gene prognostic analysis, CDKN2A, CMTM8 and ILK demonstrated the value in predicting prognosis among the 100 EMT-immune-related DEGs in TCGA dataset (Fig. 2B). The results of GEO datasets were conform to the results of above (Fig. 2C). Furthermore, the prognostic ROC curves of the three genes were established, the results showed that the AUC of CDKN2A was 0.68, 0.66, 0.58 for 1, 3, and 5 years respectively, the AUC of CMTM8 was 0.59, 0.51, 0.57 and the AUC of ILK was 0.71, 0.58, 0.60 (Fig. 2D). The correlation analysis of the above three genes with clinical and pathological indicators was conducted. The results showed the expression of CDKN2A, CMTM8, ILK were correlated with T, N, stage, vascular invasion, lymphatic invasion and microsatellite instability to some extent (Additional file 1: Figure S1A–C). To sum up, CDKN2A, CMTM8 and ILK were important genes with prognostic value in CC.

### The differential expressions of CDKN2A, CMTM8, ILK conform to their survival trend and were defined as hub genes

#### Expression analysis and validation by bulk RNA data

**Fig. 3:**
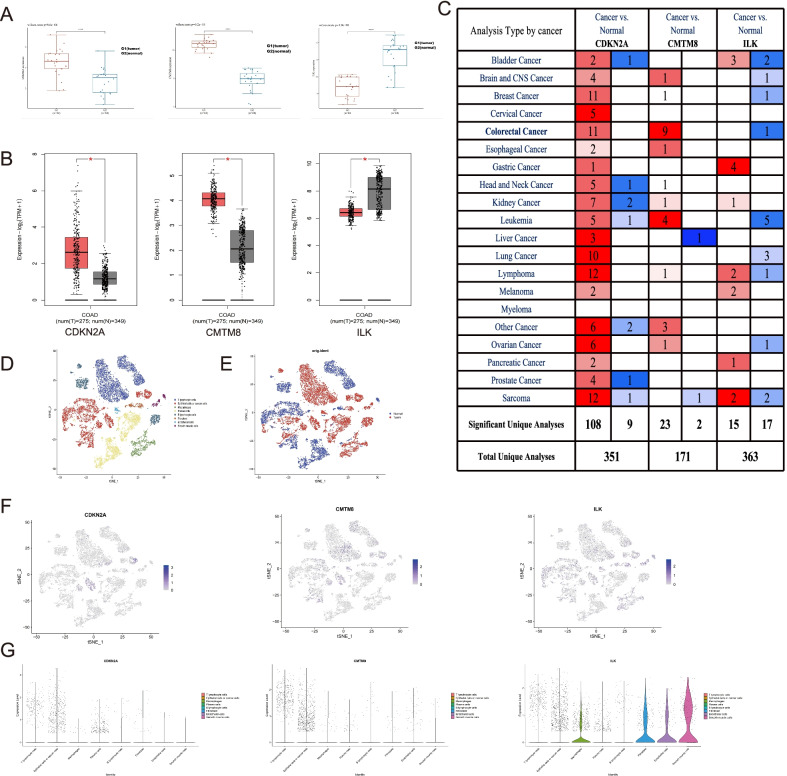
Expression analysis of CDKN2A, CMTM8 and ILK by different database and scRNA-seq data. **A** GSE10950 dataset. **B** GEPIA 2.0 database. **C** Oncomine database (*P* value < 0.05, Fold Change > 2, Gene rank = All, Data Type = mRNA). **D** Cluster diagram by cell types in the scRNA-seq data. **E** Cluster diagram by the cell origin in the scRNA-seq data. **F** The feature plots of CDKN2A, CMTM8 and ILK by scRNA-seq data. **G** The violin plots of CDKN2A, CMTM8 and ILK by scRNA-seq data

In order to analyze if the expression differences of the three genes were accord with the results of K-M curves, we conducted expression analysis by multiple datasets from different databases. The GSE10950 dataset showed that CDKN2A and CMTM8 mRNA expression levels were up-regulated in colon cancer samples, while ILK was considerably down-regulated in colon cancer samples (Fig. 3A). Through GEPIA 2.0 and Oncomine databases, the results were consistent with the GSE10950 dataset (Fig. 3B, C). By the results above, we know that CDKN2A and CMTM8 were higher expressed in cancer tissues and the prognosis will be worse when they were up-regulated. However, ILK was lower expressed in cancer tissues and the prognosis will be worse when it was down-regulated.

#### Expression validation by single-cell RNA data

As the traditional bulk profiles represents the average expression levels of the constituent cells, it does not reflect the true condition of cancer cells. Thus, verifying CDKN2A, CMTM8, ILK at the single cell resolution will increase the authenticity and reliability of the analysis results, and even improve our understanding of the underlying mechanism. So, we evaluate the expression of CDKN2A, CMTM8, and ILK in single-cell level. T cells, epithelial/tumor cells, macrophages, B cells, plasma cells, fibroblasts, endothelial cells and smooth muscle cells were annotated by single-cell RNA sequencing analysis (Fig. 3D). All cells’ distribution according to the sample sources was shown in Fig. 3E. CDKN2A and CMTM8 can be expressed in epithelial cells, tumor cells, and T cells, and the expression density was comparatively higher in colon cancer cells. ILK can be expressed in epithelial cells, tumor cells, fibroblasts, smooth muscle cells, and T cells. The expression density was relatively higher in normal epithelial cells, fibroblasts, and smooth muscle cells (Fig. 3F, G). As a result, the expression patterns could be verified by single-cell data.

#### Expression validation by immunohistochemistry wet experiments

**Fig. 4:**
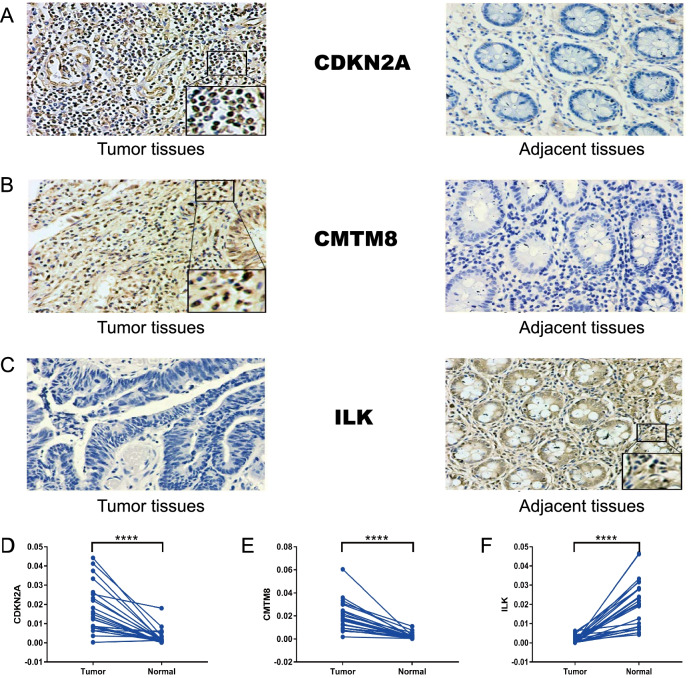
The immunohistochemistry images from wet lab. **A** The representative images of CDKN2A in the tumor and adjacent tissues. **B** The representative images of CMTM8 in the tumor and adjacent tissues. **C** The representative images of ILK in the tumor and adjacent tissues. **D** The statistical results of CDKN2A expression in the tumor and adjacent tissues. **E** The statistical results of CMTM8 expression in the tumor and adjacent tissues. **F** The statistical results of ILK expression in the tumor and adjacent tissues

In order to further verify the expression in protein level. We performed experiments in detecting protein expression of CDKN2A, CMTM8 and ILK by immunohistochemistry in the colon cancer tissues and adjacent tissues. The representative images of IHC were indicated in Fig. 4A–C. Figure 4D–F showed that the expression of CDKN2A and CMTM8 was up-regulated in tumor tissues (****, *P* < 0.0001), while the expression of ILK was down-regulated in tumor tissues (****, *P* < 0.0001). To sum up, the expression patterns of the three genes were verified by different omics and they conformed to the survival results. So, we can define CDKN2A, CMTM8, and ILK as hub genes to conduct the following analyses.

### CDKN2A, CMTM8, ILK may affect the prognosis of colon cancer patients by regulating the immune infiltration and EMT process

**Fig. 5:**
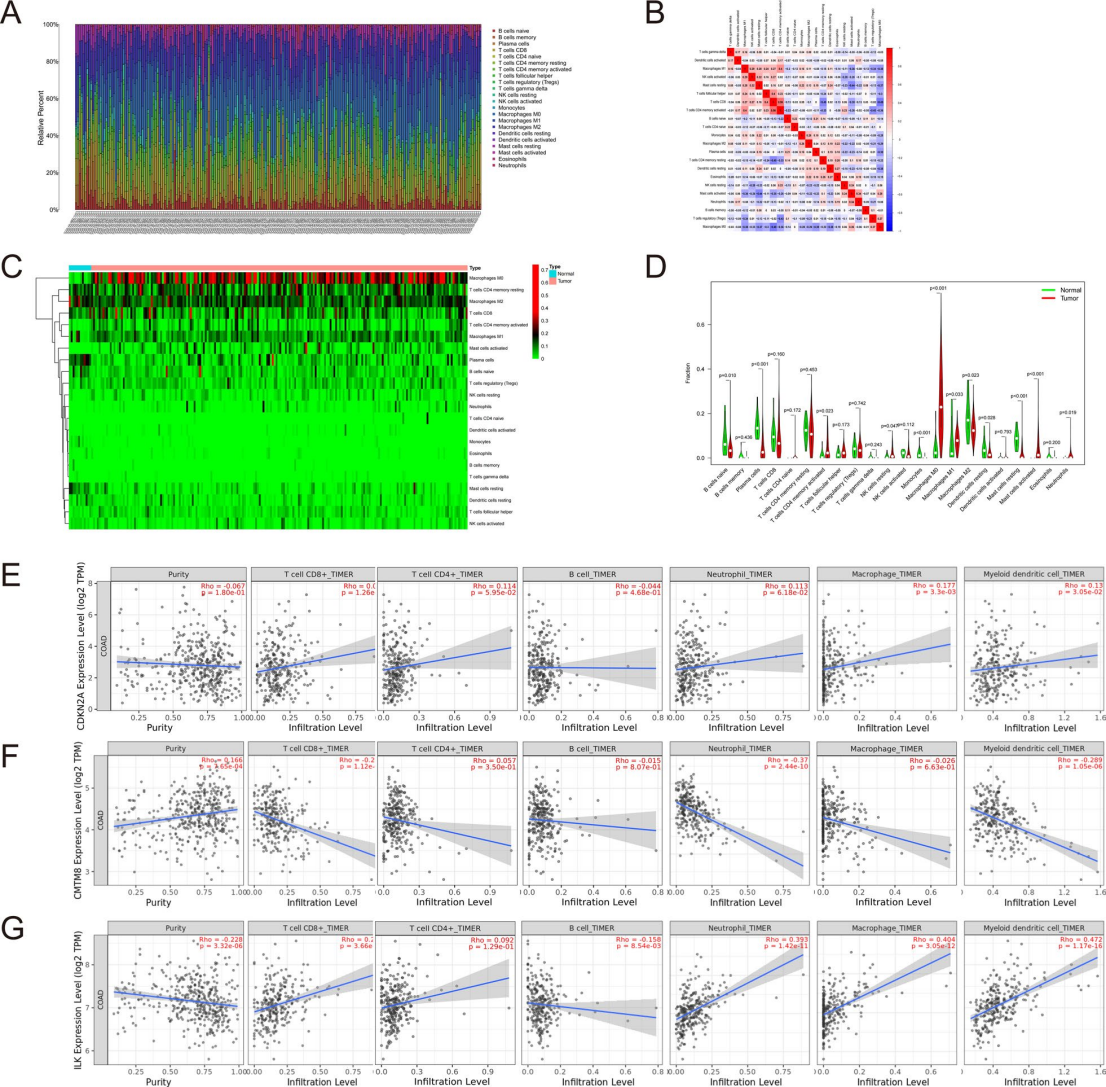
The immune related analysis in colon cancer. **A** The situation of immune cell infiltration in each TCGA-COAD sample. **B** The correlation among different immune cell types. **C** The heat plot of immune infiltration between colon and normal samples. **D** The violin plot of immune infiltration between colon and normal samples. **E** Correlation of CDKN2A expression levels with immune infiltration. **F** Correlation of CMTM8 expression levels with immune infiltration. **G** Correlation of ILK expression levels with immune infiltration

To further study how the three genes regulate the immune microenvironment, we conducted some analyses. Immune infiltration of each TCGA-COAD sample and the correlation among different immune cell types were shown in Fig. 5A, B. Figure 5C, D showed the difference in immune infiltration between CC and normal samples. The correlations between CDKN2A, CMTM8, ILK expression, and immune cell infiltrates were conducted by the TIMER algorithm. CDKN2A was positively correlated with the infiltration of macrophages (r = 0.177) and dendritic cells (R = 0.130) with statistical significance (Fig. 5E). CMTM8 was negatively correlated with CD8+ T cells (r =  − 0.231), neutrophils (r =  − 0.37), and dendritic cells (r =  − 0.289) with statistical significance (Fig. 5F). ILK was positively correlated with CD8+ T cells (r = 0.275), neutrophils (r = 0.393), macrophages (r = 0.404), dendritic cells (r = 0.472), and negatively correlated with B cells (r =  − 0.158), the results were statistically significant (Fig. 5G). Table 1 showed the correlation between the expression of CDKN2A, CMTM8, ILK and traditional immune markers. They were significantly correlated with most immune markers, and the results were largely conformed to TIMER. Overall, CDKN2A, CMTM8, ILK could regulate the immune infiltration and may result in immunosuppression to a certain degree.

**Table 1:** The expression correlation of CDKN2A, CMTM8, ILK and immune markers

Cell type	Gene markers	CDKN2A		CMTM8		ILK	
		COR	*P* value	COR	*P* value	COR	*P* value
B cell	FCRL2	− 0.008	8.77e−01	− 0.052	7.69e−01	0.077	1.22e−01
	CD19	0.05	3.18e−01	0.042	3.98e−01	0.114	2.11e−02
	MS4A1	− 0.005	9.17e−01	0.009	8.64e−01	0.062	2.15e−01
	CD22	0.117	1.84e−02	− 0.07	1.61e−01	0.202	3.96e−05
	CD70	0.17	5.88e−04	− 0.159	1.27e−03	0.332	6.64e−12
CD8+ T Cells	CD8A	0.095	5.49e−02	− 0.204	3.49e−05	0.095	5.7e−02
	CD8B	0.059	2.39e−01	− 0.041	4.04e−01	0.121	1.45e−02
Neutrophils	FCGR3B	0.105	3.44e−02	0.022	6.57e−01	0.317	5.99e−11
	CEACAM3	0.105	3.36e−01	0.151	2.3e−03	0.323	2.61e−11
	SIGLEC5	0.178	3.25e−04	− 0.124	1.26e−02	0.403	2.64e−17
	FPR1	0.159	1.28e−03	− 0.148	2.71e−03	0.417	1.75e−18
	CSF3R	0.13	8.59e−03	− 0.134	6.95e−03	0.323	2.53e−11
	S100A12	0.078	1.18e−01	− 0.053	2.86e−01	0.184	2.00e−04
Macrophages (M1)	CD68	0.144	3.56e−03	− 0.215	1.2e−05	0.301	5.81e−10
	PTGS2	0.058	2.41e−01	− 0.164	9.26e−04	0.039	4.33e−01
	IRF5	0.101	4.14e−02	0.032	5.20e−01	0.142	4.06e−03
	NOS2	− 0.028	5.75e−01	− 0.105	3.52e−02	− 0.104	3.64e−02
Macrophages (M2)	CCL2	0.179	2.79e−04	− 0.130	8.55e−03	0.348	4.93e−13
	IL10	0.179	2.64e−04	− 0.086	8.26e−02	0.304	4.11e−10
	CD163	0.175	4.09e−04	− 0.225	4.80e−06	0.326	1.77e−11
	VSIG4	0.187	1.51e−04	− 0.149	2.55e−03	0.442	7.92e−21
	CSF1R	0.155	1.78e−03	− 0.163	9.69e−04	0.422	0.422 − 19
	FCGR2A	0.124	1.74e−02	− 0.185	1.810 − 04	0.326	1.66e−11
Dendritic cells	CD209	0.084	9.15e−02	− 0.068	1.73e−01	0.189	1.23e−04
	CLEC9A	0.026	6.05e−01	0.023	6.41e−01	0.181	2.47e−04
NK cells	KIR3DL3	− 0.115	2.02e−02	− 0.061	2.19e−01	0.055	2.71e−01
	NCR1	0.026	6.07e−01	− 0.206	3e−05	0.115	2.02e−02
	KLRD1	0.085	8.8e−02	− 0.264	6.48e−08	0.208	2.44e−05
Th1 cells	TBX21	0.028	5.79e−01	− 0.185	1.71e−04	0.127	1.93e−02
Th2 cells	GATA3	0.185	1.73e−04	− 0.088	7.69e−02	0.267	4.69e−08
Treg	FOXP3	0.128	9.96e−03	− 0.15	2.48e−03	0.283	6.69e−09
	CCR8	0.059	2.36e−01	− 0.215	1.21e−05	0.224	5.03e−06
Monocyte	C3AR1	0.147	2.93e−03	− 0.168	6.76e−04	0.361	5.63e−14
	CD86	0.128	9.92e−03	− 0.206	2.76e−05	0.321	3.3e−11
	CSF1R	0.155	1.78e−03	− 0.163	9.69e−04	0.422	5.34e−19
	CD14	0.207	2.66e−05	− 0.2	5.09e−05	0.438	1.77e−20

Moreover, we also studied the influences of the three genes in regulating the expression of immune checkpoints. The results showed that CDKN2A was positively correlated with CTLA4, PD-1, TIM, LAG3. CMTM8 was negatively correlated with TIGIT, CTLA4, PD-L1, PD-1, TIM and positively correlated with LAG3. ILK was positively correlated with TIGIT, CTLA4, PD-L1, PD-1, TIM, LAG3 (Table 2). All in all, CDKN2A, CMTM8, ILK may affect the immune microenvironment by regulating the expression of immune checkpoints mentioned above. But, the study about immune microenvironment is complex and sometimes the results may confusing, which is worth further study.

**Table 2:** The expression correlation of CDKN2A, CMTM8, ILK and immune checkpoints

Immune checkpoints	CDKN2A		CMTM8		ILK	
	COR	*P* value	COR	*P* value	COR	*P* value
TIGIT	0.084	8.95e−02	− 0.252	2.71e−07	0.121	1.51e−02
CTLA4	0.103	3.77e−02	− 0.207	2.56e−05	0.106	3.22e−02
CD274 (PDL1)	0.096	5.41e−02	− 0.262	8.64e−08	0.142	4.08e−03
PDCD1 (PD1)	0.198	5.93e−05	− 0.206	2.78e−05	0.164	8.82e−04
LAG3	0.135	6.55e−03	0.222	6.38e−05	0.135	6.52e−03
HAVCR2 (TIM)	0.154	1.90e−03	− 0.216	1.10e−05	0.363	4.14e−14

We have found that CDKN2A, CMTM8, ILK may influence the prognosis of COAD patients by leading to immunosuppression. Next, in order to study how CDKN2A, CMTM8, ILK affect the EMT process, we conducted a correlation analysis between the hub genes and EMT markers. Table 3 showed that CDKN2A and CMTM8 were negatively related to the epithelial-correlated marker TJP1, while ILK was positively correlated. Mesenchyme-correlated markers, like MMP3, MMP7, MMP9, were positively related to CDKN2A and CMTM8, while ILK was negatively correlated. So, CDKN2A and CMTM8 may facilitate the EMT process, while ILK may suppress the EMT process.

**Table 3:** The expression correlation of CDKN2A, CMTM8, ILK and EMT markers

EMT markers	CDKN2A		CMTM8		ILK	
	COR	*P* value	COR	*P* value	COR	*P* value
*Mesenchymal markers*						
MMP7	0.56	9.5e−52	0.74	1.6e−107	− 0.48	6.8e−37
MMP9	0.49	8.6e−40	0.71	8.8e−96	− 0.46	1.2e−33
MMP3	0.41	3.1e−27	0.38	1.3e−12	− 0.11	0.0048
*Epithelial markers*						
TJP1	− 0.10	1.6e−02	− 0.47	8.2e−35	0.68	2.5e−85

### CDKN2A, CMTM8, ILK could affect the immune microenvironment and EMT progress by a series of pathways

**Fig. 6:**
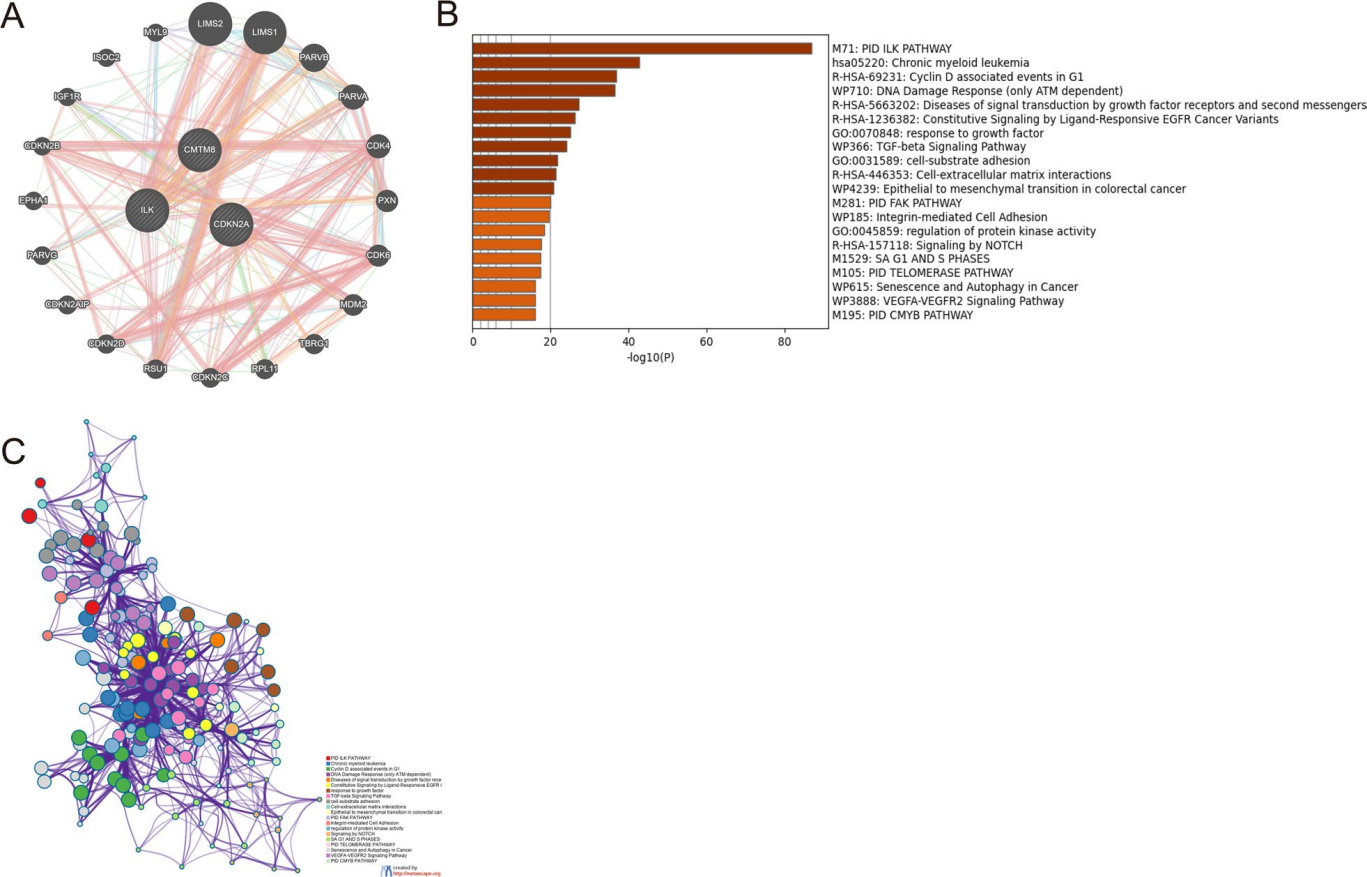
The co-expression and pathway enrichment analysis of hub genes. **A** The display of co-expressional network by GeneMANIA. **B** The pathway enrichment analysis of hub genes by Metascape. **C** The interactions among the enriched pathways

Based on the above studies, we have known that CDKN2A, CMTM8, ILK may have impact on prognosis by regulating the immune microenvironment and EMT progress. In order to further study the specific signal pathways, co-expression and pathway enrichment analyses were carried out. 130 co-expressional genes of CDKN2A, CMTM8 and ILK were obtained by the STRING database and we displayed the co-expression interactions by GeneMANIA (Fig. 6A). Then, we conducted pathway enrichment analysis of all co-expressional genes by Metascape. Figure 6B showed that the genes were concentrated in the following pathways: ILK pathway, TGF-β pathway, Epithelial to mesenchymal transition in colorectal cancer, Integrin-mediated cell adhesion, Notch pathway, and so forth. The interactions of the pathways were shown in Fig. 6C. Based on all the previous results, CDKN2A, CMTM8, ILK could affect the immune microenvironment and EMT progress by a series of signal pathways. The three hub genes may affect the prognosis of COAD patients by the above mechanisms.

### Exploring the regulatory mechanism of CDKN2A, CMTM8 and ILK

**Fig. 7:**
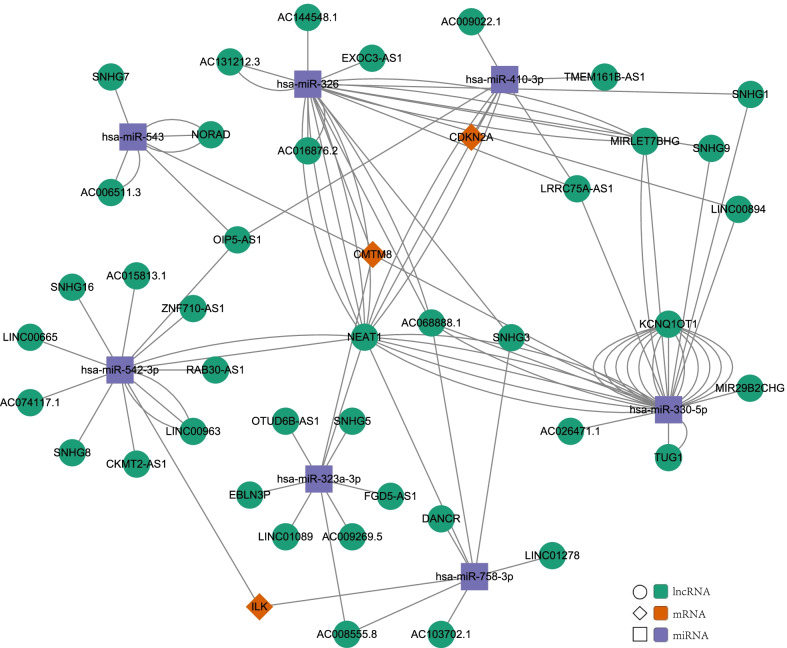
The competing endogenous RNA (ceRNA) network of lncRNA-miRNA-mRNA in colon cancer

The abnormal expression of the three hub genes may result in different overall survival rates. To clarify the expression regulatory mechanism of hub genes, the ceRNA network was established. Figure 7 showed the ceRNA network consists of 7 miRNAs and 41 lncRNAs that may mediate the expression of hub genes. So, the expression of CDKN2A, CMTM8, ILK could be regulated by ceRNAs.

Furthermore, the CNV, mutation and methylation of hub genes were analyzed. The CNV of CDKN2A was negatively correlated with the infiltration of CD8+ T cells and neutrophils (Additional file 2: Figure S2A). The infiltration of NK cells was significantly increased in CMTM8 mutation-type compared with wild-type (Additional file 2: Figure S2B). The infiltration of CD4+ T cells, neutrophils, and DC in the ILK mutation-type was significantly higher than that in the wild-type (Additional file 2: Figure S2C). The Sangerbox database was used to show mutation sites of CDKN2A, CMTM8, ILK (Additional file 3: Figure S3A). The phenotypic changes with the statistical difference between mutant samples and others showed in epithelial and EMT phenotypes, genomic changes, and microsatellite stability (Additional file 3: Figure S3B). There was no statistical difference in overall survival(OS) and disease-free survival (DFS) between mutant samples and no-mutant samples, while there was a trend of poor OS in the mutant group (Additional file 3: Figure S3C). Additional file 4: Figure S4 showed that there was no statistical difference in prognosis between methylation samples and no-methylation samples. The results above proved that the expression of the three genes could be regulated by CNV, mutation and methylation. As a result, the EMT phenotypes and immune infiltration could be changed by CNV, mutation and methylation to some extent although there was no survival difference in OS and DFS.

### The EMT-immune-related signature was an independent prognosis factor, which could better predict the prognosis

**Fig. 8:**
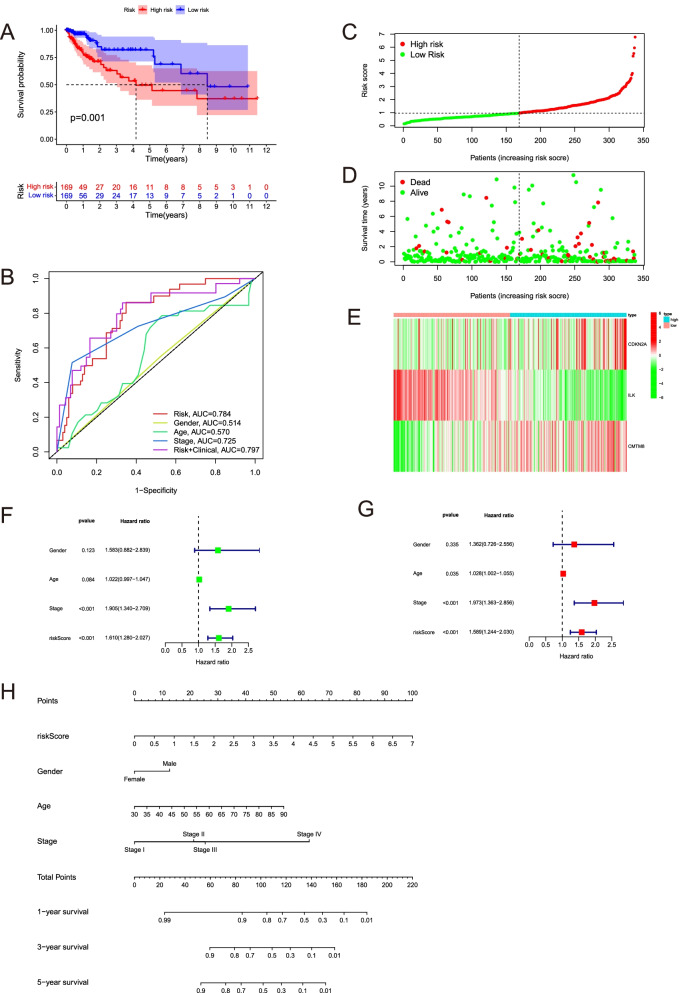
Verification the accuracy of the riskscore and predicted the survival probability of colon cancer patients combined with other clinical characters. **A** Kaplan–Meier curves between high-risk group and low-risk group by TCGA-COAD data. **B** The AUC score of the riskscore and other clinical indicators at 1 year in the TCGA-COAD data. **C** The patients from TCGA are divided into high-risk group and low-risk group based on the riskscore median values. **D** The distribution of survival time in the high-risk group and low-risk group in the TCGA data. **E** The expression heatmap of CDKN2A, CMTM8, ILK in the high-risk group and low-risk group in the TCGA data. **F** Univariate cox regression analysis of age, gender, stage, and riskscore in the TCGA data. Riskscore is significantly associated with the survival of colon cancer. **G** Multivariate cox regression analysis of age, gender, stage, and riskscore in the TCGA data. Riskscore is an independent prognostic factor for the survival of colon cancer. **H** Nomogram for the prediction of 1-, 3-, 5-year survival probability in patients with colon cancer

To better predict the prognosis of COAD patients, we built an EMT-immune-related gene signature by CDKN2A, CMTM8 and ILK with the method of multivariate Cox regression analysis. The riskscore were calculated by the following formula: 0.193446914316988 * Expression level (CDKN2A) + 0.453316103231676 * Expression level (CMTM8) − 0.667999829487826 * Expression level (ILK). Figure 8A showed that the high-risk groups were less likely to survive than the low-risk group (*p* = 0.001). ROC curves showed the AUC scores of the riskscore in 1-years were 0.784. If we combined the riskscore with other clinical indicators, the AUC score can reach 0.797(Fig. 8B). Figure 8C displayed that all patients from TCGA-COAD were divided into high-risk and low-risk groups. Figure 8D showed the distribution of survival time in the high-risk group and low-risk group. The expression of hub genes in the high-risk group and low-risk group were shown in the heatmap (Fig. 8E). By the univariate and multivariate Cox regression analysis, we found the riskscore was an independent prognosis factor (Fig. 8F, G). Nomogram is significant to predict prognosis, our nomogram includes the riskscore, gender, age, stage (Fig. 8H). The calibration curves were shown in Additional file 5: Figure S5. The signature we constructed could predict the prognosis of colon cancer better than a single gene.

## Discussion

An accumulating number of studies have shown that EMT and immunosuppression could appear together in multiple tumors. This phenomenon could promote the metastasis of the tumor and finally affect prognosis [10]. Some researchers have conducted primary explorations on biomarkers related to EMT and immune infiltration. The results demonstrated the significant role of the EMT-immune-related biomarkers in prognosis predicting, and they could be seen as potential therapeutic targets. However, more biomarkers should be identified. To further explore prognostic biomarkers associated with EMT and immune infiltration, we conducted differential expression analysis by GSE10950 data and TCGA&GTEx data. 100 differentially expressed EMT-immune-related genes were screened out. By prognostic analysis, CDKN2A, CMTM8 and ILK demonstrated the value in predicting prognosis. CDKN2A, CMTM8, ILK could be seen as potential prognostic biomarkers in CC and deserve further studies.

Cyclin-Dependent Kinase Inhibitor 2A (CDKN2A) is a significant gene belonging to the family of cyclin-dependent kinase inhibitor genes, which serves a regulatory role in cell proliferation and apoptosis [43]. Recent studies have found that CDKN2A is positively correlated with LIPH expression and affects the EMT process [17]. Moreover, CDKN2A is related to immune infiltration [44]. As a result, combined with our analysis, CDKN2A may take part in the process of affecting EMT and immune. A study revealed that CDKN2A is the target of quercetin in reducing death caused by COAD [45]. Another study showed that CDKN2A, as one of the 8 autophagy genes prognostic signature in colon cancer, can be seen as a good biomarker [46]. These are consistent with our results and prove the prognostic value of CDKN2A in CC. Nowadays, the CDKN2A gene has been rarely studied in colon cancer. However, many studies have found that CDKN2A is related to poor prognosis in bladder cancer, pancreatic adenocarcinoma, hepatocellular carcinoma (HCC), and so on [47–49]. Among these studies, Luo et al. found that CDKN2A is highly expressed in HCC and associated with poor overall survival and disease-free survival. They also found that the expression of CDKN2A is associated with immune cells infiltration. Another study showed that CDKN2A may lead to the downregulation of the immune response and enhance the recurrence risk of metastatic colorectal cancer [50]. We found that CDKN2A is up-regulated in CC and result in poor OS. Moreover, CDKN2A may lead to immunosuppression by affecting immune infiltration and the expression of immune checkpoints. We found that CDKN2A has a stronger association with M2 subtype macrophages and it shows a positive correlation. We also found that the CD8+ T cell infiltration will decrease when the copy number of CDKN2A increases. It could explain why CDKN2A may influence the immune microenvironment and the prognosis of patients. Our results conform to the previous studies. In this study, CDKN2A was negatively correlated with epithelial-related markers and positively correlated with mesenchyme-related markers. This possibly suggests the promoting effect of CDKN2A on EMT progression to some extent. It’s consistent with the findings from Zhuang et al. [17]. To sum up, CDKN2A is up-regulated in CC and result in a poor prognosis. This phenomenon may be caused by two potential mechanisms, one is immunosuppression and the other is the progression of EMT.

The CKLF-like MARVEL transmembrane domain-containing (CMTM) family regulates aggressive phenotype in many cancers, which consists of 8 member proteins (CMTM1-8). The roles of CMTM members in different cancers are largely unknown [51, 52]. Some studies have found that CMTM8 is associated with EMT and the immune system [53, 54]. Although CMTM8 was rarely studied in colon cancer before, previous researches of other fields could give us some guides. Lu et al. contend that the CMTM family is highly correlated with tumor and immune diseases, and has important value in diagnosis and treatment [55]. Some researchers have shown that the deletion of CMTM8 can inhibit the migration and invasion of pancreatic cancer, whereas high expression of CMTM8 can promote migration and invasion. The interaction between CMTM8 and LPA1 leads to activation of oncogenic Wnt-β-catenin signalling [52]. By facilitating the Wnt-β-catenin signaling pathway, CMTM8 may induce EMT and tumor growth and metastasis [56]. We found that CMTM8 is highly expressed in colon cancer and leads to a poor prognosis. Furthermore, CMTM8 is negatively correlated with epithelial-related markers and positively correlated with mesenchyme-related markers. This possibly suggests the promoting effect of CMTM8 on EMT progression to some extent. These results are consistent with former researches. In another hand, our immune infiltration related studies suggested that CMTM8 may affect the functions of immune killing and antigen presentation, which finally leads to immunosuppression. The previous analysis about the relation of CMTM8 and immune infiltration is little, so it deserves more studies in the future to verify our results. In summary, CMTM8 is up-regulated in CC and result in a poor prognosis. It may be caused by immunosuppression and the progression of EMT. But a research demonstrated that CMTM8 is low expressed in tumors, for example, lung, gastrointestinal tract, and so on [53]. Another study showed that CMTM8 is down-regulated in gastric cancer, which is associated with poor prognosis [57]. Notably, some contended that the CMTM family is complex that could exert either a positive or negative effect on tumor cells. CMTM research is still in its infancy and needs further studies [54]. The exact effect of CMTM8 in colon cancer needs additional analysis and validation.

Integrin-linked kinase (ILK) plays a key role as a multifunctional effector of growth factor signaling and cell–matrix interaction [58]. Some studies have found that ILK is associated with the process of EMT and immune, which could be seen as an EMT-immune-related gene [59, 60]. A study found that ILK is up-regulated in colorectal cancer and other tumors [60]. We found that ILK is down-regulated in colon cancer and leads to a poor prognosis. The previous studies were not consistent with our findings. The expression of ILK have been validated by multiple datasets in this study, for example, GSE10950 from GEO database, TCGA and GTEx datasets from GEPIA database, Oncomine database. Then, we also used a single-cell RNA sequencing dataset and the results were conform to the expression trend of the bulk data. Furthermore, we conducted immunohistochemistry experiments to do further validation in protein level. All the results above could support the results of expression of ILK. So we contend that ILK may be a relatively complex gene in cancer and need further studies in the future. Huang et al. found that down-regulated PARVA in prostate cancer might promote cancer progression through releasing the inhibition of ILK activity and consequently upregulating the MAPK/ERK pathway [61]. As a result, ILK could act as a tumor suppressor gene to affect the prognosis of tumors, which is consistent with our findings. Furthermore, we found that ILK may affect the function of immune infiltration and antigen presentation, and eventually promote immune killing. ILK may not only positively associated with M2 subtype macrophages, but also M1 subtype macrophages. So it’s influence on immune microenvironment may not depend on macrophages alone, but also CD8+ T cells, neutrophils, dendritic cells. On the other hand, ILK may suppress EMT, because it was positively correlated with epithelial-related markers and negatively correlated with mesenchyme-related markers. So, ILK is down-regulated in CC and result in a poor prognosis. This phenomenon may be caused by two potential mechanisms, one is immunosuppression and the other is the progression of EMT. However, we should know that the function of ILK in CC is complex, so its exact effect needs further analysis and validation.

To sum up, we found that CDKN2A and CMTM8 were up-regulated in colon cancer, while, ILK was down-regulated. We verified the expression in different omics. Moreover, 338 colon cancer patients from TCGA were enrolled in the prognostic analysis. We found that CDKN2A and CMTM8 were tumor promoters, while ILK was tumor inhibitors. Therefore, the results of this study were credible to some extent. The difference from the previous studies may be related to the complex functions of the genes, distinct ways of getting specimens, different experiment methods, and the bias of samples. Immune microenvironment is complex and could be affected by many aspects. For example, immune cell infiltration, immune checkpoint regulation, antigen presentation process and so forth. The study about immune microenvironment is complex and sometimes the results may contradictory. These all worth further studies.

CDKN2A, CMTM8, ILK, and their co-expression genes were enriched in the following pathways: ILK pathway, TGF-β pathway, epithelial to mesenchymal transition in colorectal cancer, integrin-mediated cell adhesion, signaling by Notch, and so on. These results not only proved their effect on the EMT process but also further studied the possible pathways to affect the EMT process. It is worth noting that a large number of studies have found that TGF-β pathway activation can not only induce EMT development but also induce immunosuppression [10, 62]. Combined with our results, it can be inferred that the up-regulated CDKN2A, CMTM8, and down-regulated ILK may aggravate the progression of EMT by the TGF-β pathway in colon cancer, as a result, promoting the infiltration and metastasis. Besides, CDKN2A, CMTM8 and ILK may affect intercellular signal transduction through the TGF-β pathway. Then they could result in abnormal immune infiltration, expression of the immune checkpoints, and antigen presentation function. Eventually, this phenomenon will lead to the formation of the immunosuppressant microenvironment. So, CDKN2A, CMTM8 and ILK may affect the prognosis of colon cancer by influencing EMT and immune simultaneously. However, TGF-β may not be the only signaling pathway in these processes. Previous studies have shown that the Notch pathway is not only involved in the induction of EMT [63, 64] but also regulates the immune microenvironment and leads to the occurrence of immune escape [65–67]. In conclusion, the three genes may affect the progression of immune infiltration and EMT by the TGF-β and the Notch pathway.

miRNA and lncRNA could exert the important function of regulating gene expression, and their regulatory networks are involved in many biological processes. We think ceRNA is important for helping us to find the targets and pathways for colon cancer. As a result, this analysis lays a foundation for our subsequent research. In general, the expression of CDKN2A, CMTM8 and ILK could be regulated by CNV, mutation and methylation. As a result, the EMT phenotypes and immune infiltration may be changed to some extent. However, there was no survival difference in OS and DFS based on CNV, mutation and methylation. The specific effects and corresponding mechanisms need to be further studied by expanding sample size and conducting basic experiments.

At last, we established an EMT-immune-related signature using CDKN2A, CMTM8, ILK. This signature was an independent prognosis factor in colon cancer, which could predict the prognosis better. A nomogram has been built using the riskscore, gender, age and stage. It could give us important guidance in predicting the survival rate of colon cancer in the clinic.

There are some limitations in the current study, and it is necessary to do further analysis to verify the results, for example, stain the tumor slides with EMT markers and immune markers. And we will use our own samples in subsequent experiments to verify the prognostic value of the three genes. Futhermore, the studies about immune microenvironment and so on are complex and sometimes the results may confusing. So, further experiments are needed to study the function of genes and the specific mechanisms affecting EMT and immune function.

## Conclusions

In conclusion, CDKN2A, CMTM8 and ILK could be involved in promoting the process of EMT and forming the immunosuppression microenvironment through the TGF-β pathway, the Notch pathway, and so forth. As a result, the three genes could significantly affect the prognosis of colon cancer patients ultimately. CDKN2A, CMTM8 and ILK are valuable prognostic factors and potential therapeutic targets, which need further study

## Supplementary Information

**Additional file 1: Figure S1**. The correlative analysis with clinical indicators (T, N, M, AJCC Stage, venous or lymphatic invasion and microsatellite instability). (A) CDKN2A. (B) CMTM8. (C) ILK.

**Additional file 2: Figure S2**. The correlation between the CNV or mutation of hub genes and the immune infiltration. (A) CDKN2A. (B) CMTM8. (C) ILK.

**Additional file 3: Figure S3**. The mutation analysis of hub genes. (A) The mutation sites of CDKN2A, CMTM8, ILK by sangerbox database. (B) The phenotypic changes with statistical difference between mutant and no-mutant sanmples. (C) The difference of OS and DFS between mutant samples and no-mutant samples.

**Additional file 4: Figure S4**. The effect of methylation in prognosis of colon cancer. (A) CDKN2A. (B) CMTM8. (C) ILK.

**Additional file 5: Figure S5**. The calibration curves of the nomogram for colon cancer patients in the TCGA data. (A) Calibration curve for 1-year OS. (B) Calibration curve for 3-year OS. (C) Calibration curve for 5-year OS.

## Data Availability

The datasets GSE166555, GSE10950, GSE39582, GSE24511 and GSE29621 are available in the GEO database (http://www.ncbi.nlm.nih.gov/geo). Meanwhile, the TCGA repository (https://portal.gdc.cancer.gov/projects/TCGA) was utilized, under the accession code: Colon adenocarcinoma (COAD). GTEx data were used by GEPIA online database.

## Abbreviations

CC: Colon cancer
scRNA-seq: Single-cell RNA sequencing
EMT: Epithelial-mesenchymal transition
TCGA: The Cancer Genome Atlas
COAD: Colon adenocarcinoma
IHC: Immunohistochemistry
DEGs: Different expression genes
ROC curves: Receiver operating characteristic curves
