# Supplementary figures and images for: Identification and validation of EMT-immune-related prognostic biomarkers CDKN2A, CMTM8 and ILK in colon cancer
Source: BMC Gastroenterol. 2022 Apr 16;22:190. doi: 10.1186/s12876-022-02257-2 (PMC9013447; doi:10.1186/s12876-022-02257-2)

A

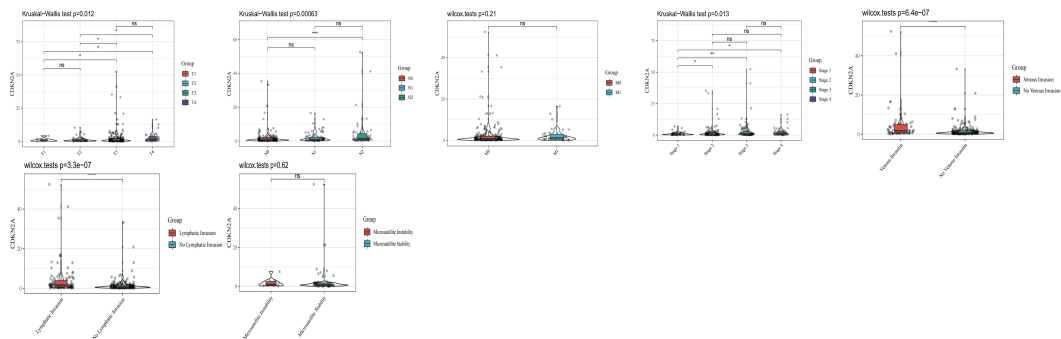

B

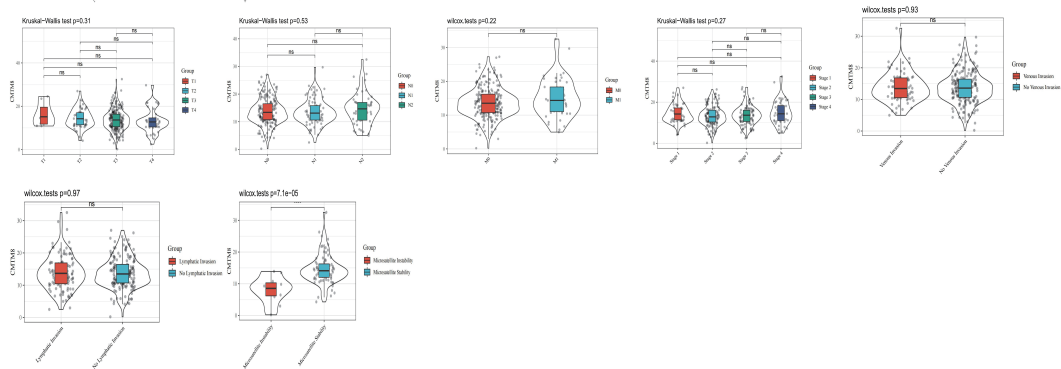

C

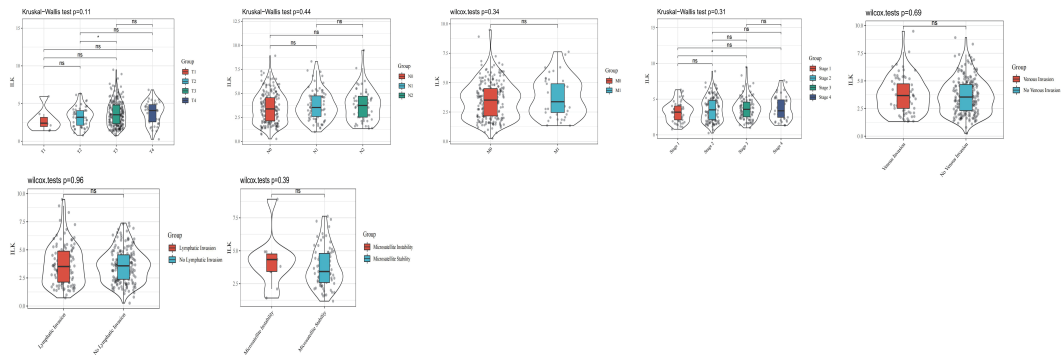

Supplement: Supplementary file 1 — Additional file 1: Figure S1. The correlative analysis with clinical indicators (T, N, M, AJCC Stage, venous or lymphatic invasion and microsatellite instability). (A) CDKN2A. (B) CMTM8. (C) ILK. [file 12876_2022_2257_MOESM1_ESM.pdf]

B

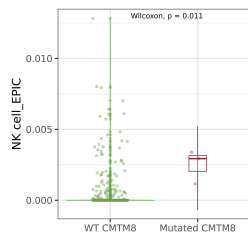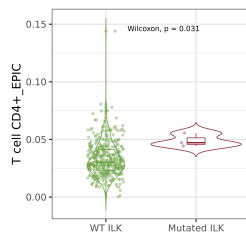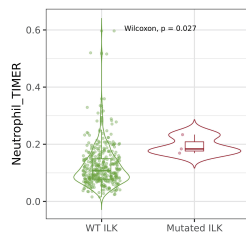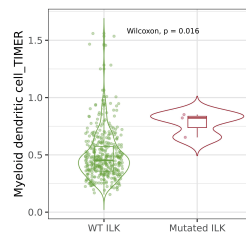

Supplement: Supplementary file 2 — Additional file 2: Figure S2. The correlation between the CNV or mutation of hub genes and the immune infiltration. (A) CDKN2A. (B) CMTM8. (C) ILK. [file 12876_2022_2257_MOESM2_ESM.pdf]

A

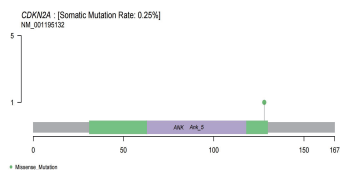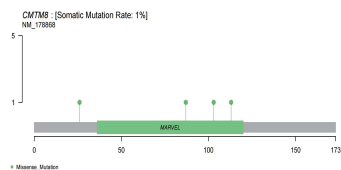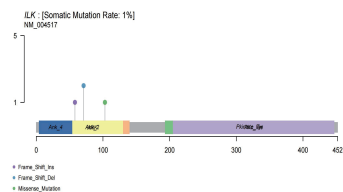

B

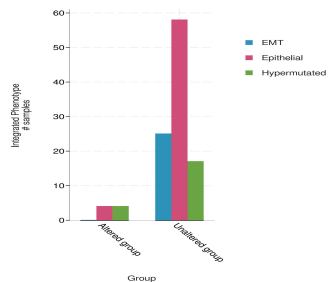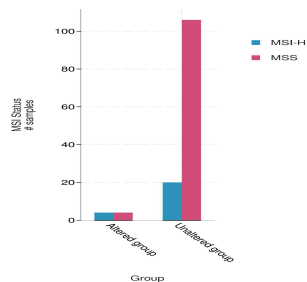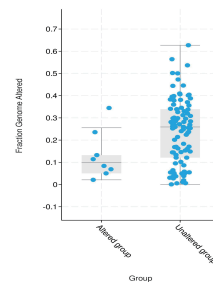

C

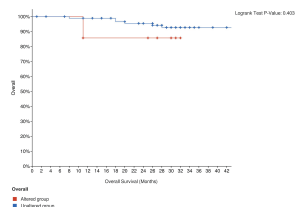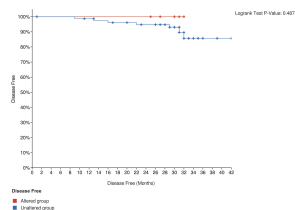

Supplement: Supplementary file 3 — Additional file 3: Figure S3. The mutation analysis of hub genes. (A) The mutation sites of CDKN2A, CMTM8, ILK by sangerbox database. (B) The phenotypic changes with statistical difference between mutant and no-mutant sanmples. (C) The difference of OS and DFS between mutant samples and no-mutant samples. [file 12876_2022_2257_MOESM3_ESM.pdf]

A

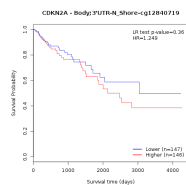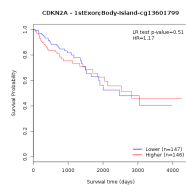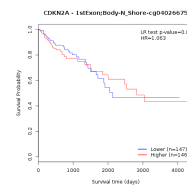

B

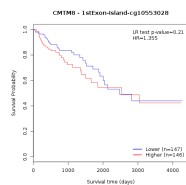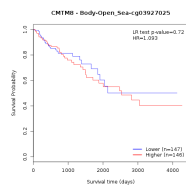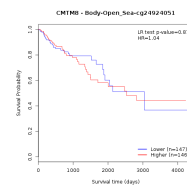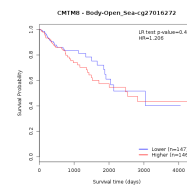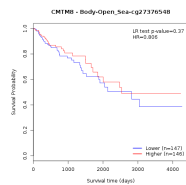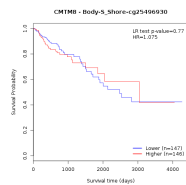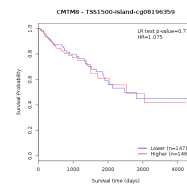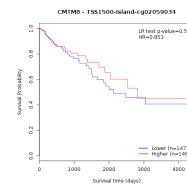

C

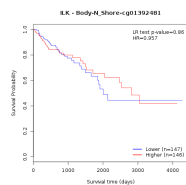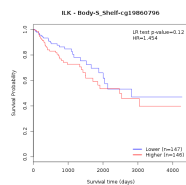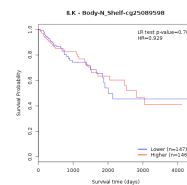

Supplement: Supplementary file 4 — Additional file 4: Figure S4. The effect of methylation in prognosis of colon cancer. (A) CDKN2A. (B) CMTM8. (C) ILK. [file 12876_2022_2257_MOESM4_ESM.pdf]

A

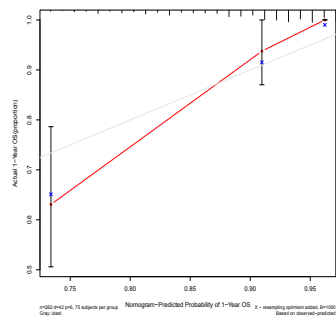

B

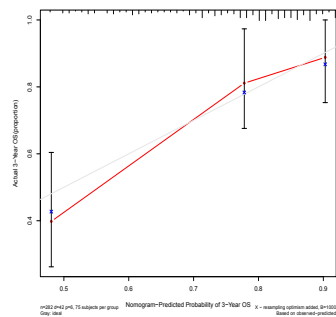

C

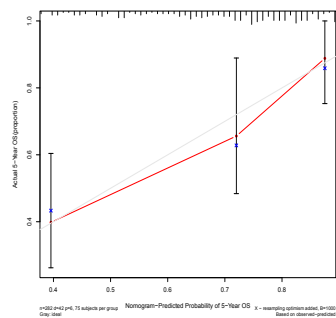

Supplement: Supplementary file 5 — Additional file 5: Figure S5. The calibration curves of the nomogram for colon cancer patients in the TCGA data. (A) Calibration curve for 1-year OS. (B) Calibration curve for 3-year OS. (C) Calibration curve for 5-year OS. [file 12876_2022_2257_MOESM5_ESM.pdf]
